# Evaluation of antioxidant profile of various solvent extracts of *Carissa opaca* leaves: an edible plant

**DOI:** 10.1186/s13065-017-0300-6

**Published:** 2017-08-18

**Authors:** Sumaira Sahreen, Muhammad Rashid Khan, Rahmat Ali Khan

**Affiliations:** 1 Botanical Sciences Division, Pakistan Museum of Natural History, Garden Avenue, Shakarparian, Islamabad, Pakistan; 2 Department of Biochemistry, Faculty of Biological Sciences, Quaid-i-Azam University Islamabad, Islamabad, Pakistan; 3 Department of Biotechnology, Faculty of Biological Sciences, University of Science and Technology, Bannu, 28100 KPK Pakistan

**Keywords:** *Carissa opaca* leaves, Free radical scavenging, Solvent–solvent extraction, Total phenolics and flavonoids

## Abstract

**Background:**

*Carissa opaca* leaves were conventionally recommended by local hakims in Pakistan for curing various human diseases including renal, hepatic and jaundice. In this work we arranged to study the antioxidant status of various fractions of *C. opaca* leaves through nine multifaceted assay systems.

**Methods:**

Various fractions were prepared through solvent–solvent extraction technique on the basis of their polarity. The fractions were screened via different free radicals viz; DPPH·, ABTS^·+^,OH·, O_2_·, iron chelating and hydrogen peroxide assays. Total concentrations of phenolic content (TPC) and flavonoids were studied.

**Results:**

Various fractions of *C. opaca* leaves showed significant activities against the tested reactive free radicals. The *C. opaca* was shown to have the highest TPCs with lowest EC_50_ values for the DPPH·, ABTS^·+^ radical scavenging capacities and iron chelating scavenging efficiency, moreover, *C. opaca* had best activities in scavenging of superoxide radicals and hydrogen peroxide as well as potently scavenged the hydroxyl radicals.

**Conclusion:**

These results suggest the potential of *C. opaca* leaves as a medicine against free-radical-associated oxidative damage.

## Background

Plants are well-known excellent perspectives for the discovery of new therapeutical products. In recent years, an ample interest has been developed in finding natural antioxidants from commonly available wild plants, fruits and vegetables that were generally mistreated [1–3]. It is believed that they possess a remarkable potential to overwhelm the deadly diseases of modern world. Numerous reports of crude extracts and pure natural compounds have been appeared for antioxidant and radical-scavenging activities [4–7]. Phenolic compounds which are secondary metabolites in plants are one of the most widely occurring groups of phytochemicals that exhibit antiallergenic, antimicrobial, antiartherogenic, antithrombotic, anti-inflammatory, vasodilatory and cardio protective effects [8, 9]. Due to the presence of the conjugated ring structures and hydroxyl groups; many phenolic compounds have the potential to function as antioxidants by scavenging or stabilizing free radicals involved in oxidative processes through hydrogenation or complexing with oxidizing species that are much stronger than those of vitamins C and E [10, 11].

*Carissa opaca* Stapf ex Hanes, is a 2–3 m tall evergreen shrub containing glabrous or puberulous branches with opposite and ovate glabrous leaves, hard and sharp spines arising between the petiole. Flower color white with 12 mm long slender corolla tube. Edible berry fruits with dark purple color after ripening. Distribution of plant in Pakistan is from Punjab to Himalayas up to 6000 ft, in Murree. The leaves are used traditionally for the treatment of asthma, cardiac dysfunction, hepatitis and jaundice. Due the lack of scientific studies of its potential pharmacological properties, the objective of this study was to evaluate the antioxidant activity through direct free radical scavenging methods and also elucidate total phenolic content (TPC) and polyphenolic flavonoids constituents of various fractions of *C. opaca* leaves.

## Results

### Total phenolics, total flavonoids and % yield contents (TPC)

Content of phenolics compounds, flavonoids and % yield contents in various fractions are exhibited in Table 1. The % yield extractions are in descending order of methanol > chloroform > ethyl acetate > *n*-hexane showing that methanol possesses a significant high amount of % yield contents. Table 1 also summarizes that methanolic extract have the highest total phenolic (*P* < 0.01) (332 ± 1.53 mg GAE/g dry extract) and (11.4 ± 0.45 mg rutin/g dry extract) in comparison with other fractions of SA extract.

**Table 1:** Antioxidant effect (EC_50_) on DPPH radicals, superoxide radicals, total antioxidant capacity and hydroxyl radicals of methanol extract and soluble fractions of *C. opaca* leaves

Plant extracts	EC_50,_ µg/ml			
	Scavenging ability on DPPH radicals	Scavenging ability on superoxide radicals	Total antioxidant capacity	Scavenging ability on hydroxyl radicals
MLC	58 ± 1.6c	93 ± 1.92b	30 ± 1.5b	22 ± 1.4b
HLC	358 ± 4.92e	135 ± 3.6c	>250e	22 ± 1.3b
ELC	444 ± 4.11f	206 ± 4.23e	>250e	18 ± 0.7a
CLC	>500g	132 ± 3.6c	>250e	18 ± 1.1a
BLC	170 ± 2.7d	229 ± 5.4f	156 ± 3.9d	18 ± 0.9a
ALC	38 ± 1.33b	159 ± 2.45d	81 ± 2.7c	18 ± 0.89a
Ascorbic acid	16 ± 1.6a	21.86 ± 1.3a	22 ± 1.8a	30 ± 1.1c
Rutin	18 ± 1.19a	–	–	–

–, Not determined

Each value in the table is represented as mean ± SD (n = 3)

Values in the same column followed by a different letter are significantly different (p < 0.05)

### Invitro antioxidant activities

**Fig. 1:**
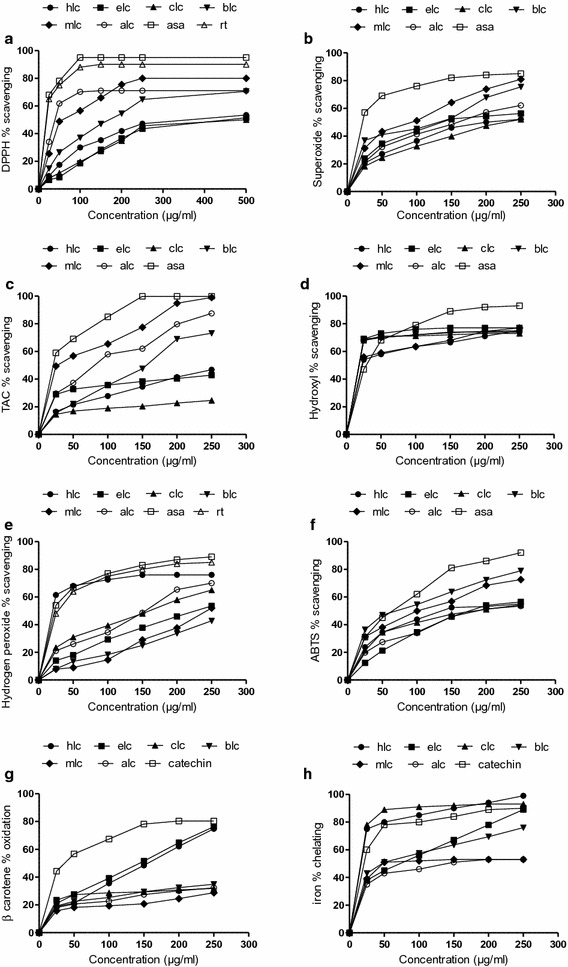
Scavenging potential of *C. opaca* leaves. **a** DPPH radical scavenging activity. **b** Superoxide inhibition. **c** Total antioxidant capacity. **d** hydroxyl percentage inhibition. **e** Hydrogen peroxide percent inhibition. **f** ABTS percent inhibition. **g** β-Carotene percent inhibition. **h** Chelating ability of various fractions of *C. opaca* fruit at different concentrations. Each value represents a mean ± SD (n = 3). *hlc n*-Hexane fraction, *elc* ethyl acetate fraction, *clc* chloroform fraction, *blc* butanol fraction, *mlc* methanol extract, *alc* aqueous fraction, *rt* rutin, *asa* ascorbic acid

DPPH radical has been widely used to assess the antioxidative activity of plant extracts. Figure 1a shows that the scavenging effect of different fractions on DPPH radical was in the following order: ALC (*C. opaca* leaves aqueous extract) >MLC (*C. opaca* leaves methanol extract) >BLC (*C. opaca* leaves butanol extract) >HLC (*C. opaca* leaves *n*-hexane extract) >EFC (*C. opaca* leaves ethyl acetate extract) and CLC (*C. opaca* leaves chloroform extract) fractions (Table 1). The superoxide radical scavenging effect of various fractions were compared with the same doses of ascorbic acid ranging from 25 to 250 µg/ml as shown in Fig. 1b. In fact, EC_50_ values in superoxide scavenging activities were in the order of MLC > CLC > HLC > ALC > ELC and BLC (Table 1). Figure 1c depicts the total antioxidant capacity of different fractions of MLC that can be ranked in the order of MLC > ALC > BLC > HLC > ELC and CLC. The EC_50_ value of antioxidant capacity for the MLC, ALC and BLC was 30 ± 1.5 µg/ml, 81 ± 2.7 µg/ml and 156 ± 3.9 µg/ml, respectively, while for the rest of the fractions EC_50_ was >250 µg/ml (Table 1). In this present investigation, the EC_50_ value of hydroxyl radical scavenging activity of ELC, CLC, BLC and ALC was 18 ± 0.7; 18 ± 1.1; 18 ± 0.9; 18 ± 0.89 µg/ml while for MLC and HLC fractions was 22 ± 1.4; 22 ± 1.3 µg/ml (Table 1). In current study, antioxidant potential of all the fractions of *C. opaca* leaves was significantly higher than that of reference compound. This situation has created a certainty for analyzing naturally occurring antioxidant substances which may be used in place of synthetic antioxidants (Fig. 1d).

**Fig. 2:**
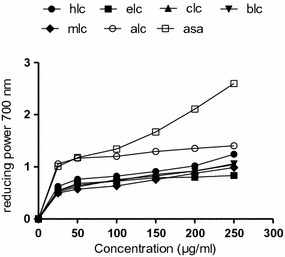
Reducing power of various fractions of *C. opaca* leaves at different concentrations. Each value represents a mean ± SD (n = 3). *hlc n*-hexane fraction, *elc* ethyl acetate fraction, *clc* chloroform fraction, *blc* butanol fraction, *mlc* methanol extract, *alc* aqueous fraction, *rt* rutin, *asa* ascorbic acid

The scavenging effect of methanol and its different fractions on hydrogen peroxide was concentration-dependent (25–250 µg/ml) as shown in Fig. 1e. As compared with the EC_50_ values, the hydrogen peroxide-scavenging activity of HLC fraction was 19 ± 1.1 µg/ml and was more effective than that of rest of the fractions as well as ascorbic acid (Table 2). The ability to scavenge hydrogen peroxide radicals of various solvent extracts from *C. opaca* leaves was in the order of HLC > MLC > CLC > ELC > ALC and BLC. Figure 1f shows that the ABTS radical scavenging ability of samples can be ranked as BLC > MLC > HLC > ELC > CLC and ALC. The EC_50_ values obtained for the BLC (70 ± 3.2 µg/ml) was significantly different (p < 0.05) from the EC_50_ values obtained for the ALC (187 ± 3.8 µg/ml), which were comparable (Table 2) with reference compound. The antioxidant activity with regard to the β-carotene bleaching assay of extract of *C. opaca* leaves can be ranked as ELC > HLC > MLC > CLC > BLC and ALC. β-Carotene bleaching assay showed the dose response curve for all the fractions at concentrations ranging from 25–250 µg/ml (Fig. 1f). The EC_50_ values of ELC and HLC were 145 ± 4.3 and 157 ± 3.12 µg/ml, respectively (Table 2) which was comparable with catechin. This data suggested that ELC and HLC fractions have a notable ability to react with free radicals to convert them into more stable non-reactive species and to terminate radical chain reaction. Figure 1h shows that all fractions were better ferrous ion chelators. The chelating activity was correlated well with the increasing concentration of each sample. The sequence for chelating power was HLC > CLC > MLC > BLC > ELC > AFC. The iron chelating data measured at different concentrations (25–250 µg/ml) suggested that ferrous ion chelating effects of all the fractions of *C. opaca* leaves would be rather beneficial to protect against oxidative damage. The EC_50_ values of iron chelating activity for various fractions are presented in Table 2. Increasing absorbance at 700 nm indicates an increase in reducing ability. Figure 2 shows the dose–response curves for the reducing powers of all extracts (25–250 µg/ml) from *C. opaca* leaves. It was found that the reducing power increased with concentration of each sample. The ranking order for reducing power was ALC > HLC > BLC > CLC > MLC > ELC. The MLC exhibited a good reducing power of 1.405 ± 0.14 at 250 µg/ml may be attributed to the collective antioxidant effects of phenolics and flavonoid.

**Table 2:** Antioxidant effect (EC_50_) on hydrogen peroxide radicals, ABTS radicals, inhibition of β carotene and chelating power of methanol extract and soluble fractions of *C. opaca* leaves

Plant extracts/chemical	EC_50,_ µg/ml			
	Scavenging ability on hydrogen peroxide radicals	Scavenging ability on ABTS radicals	β-carotene bleaching inhibition	Chelating power
MLC	155 ± 3.2b	104 ± 4.6b	>250c	49 ± 1.9b
HLC	19 ± 1.1a	133 ± 3.5c	157 ± 3.12b	16 ± 0.98a
ELC	225 ± 6.39c	176 ± 4.0d	145 ± 4.3b	73 ± 2.9c
CLC	160 ± 4.7b	181 ± 3.1d	>250c	16 ± 1.1a
BLC	>250d	70 ± 3.2a	>250c	50 ± 2.3b
ALC	243 ± 2.5c	187 ± 3.8d	>250c	137 ± 3.76d
Ascorbic acid	23.04 ± 1.7a	67 ± 2.5a	–	–
Catechin	–	–	38 ± 2.8a	20 ± 1.2a
Rutin	29.04 ± 1.5a	–	–	–

–, Not determined

Each value in the table is represented as mean ± SD (n = 3)

Values in the same column followed by a different letter are significantly different (p < 0.05)

### Correlation of EC_50_ values of antioxidant activities and phytochemical contents

Through correlation analysis for phytochemical contents with EC_50_ values of radical scavenging activity of various soluble fractions of *C. opaca* leaves and the contents of phenolics and flavonoids, non-significant correlation was found between the total phenolics and flavonoids and the antioxidant activity of various fractions (Table 3).

**Table 3:** Correlations between the EC_50_ values of antioxidant activities and phenolic and flavonoids content of *C. opaca* leaves

Assays	Correlation R^2^	
	Phenolics	Flavonoids (ns)
EC_50_ of DPPH radical scavenging ability	0.1774ns	0.5133
EC_50_ of superoxide radical scavenging ability	0.628a	0.1421
EC_50_ of antioxidant capacity	0.175ns	0.3276
EC_50_ of hydroxyl radical scavenging ability	0.4215ns	0.3649
EC_50_ of hydrogen peroxide radical scavenging ability	0.3411ns	0.1284
EC_50_ of ABTS radical scavenging ability	0.0033ns	0.2154
EC_50_ of β-carotene bleaching inhibition	0.1191ns	0.0953
EC_50_ of chelating power	0.0084ns	0.0079

*C. opaca* leaves methanol extract and its soluble fractions were used in the correlation

Significantly different depicts that a (p < 0.05), ns (non-significant)

## Discussion

*Carissa opaca* leaves is used ethno pharmacologically for the treatment of various complaints. The therapeutic benefit of medicinal plants is usually contributed to their antioxidant properties. The biochemical investigation reported that *C. opaca* leaves constitute of antioxidant compounds such as carotenoids, catechin, rutin, quercetin and other phenolics [12, 13]. Moreover, *C. opaca* leaves activities against oxidative stress, antibacterial and antitumor were yet to be explored. Different free-radical generating systems were used to assess the free-radical scavenging and reducing properties of the crude polar and non-polar extracts of *C. opaca* leaves along with evaluation of the total phenolic content. Quantitative estimation proved that the *C. opaca* leaves possesses the highest concentration of phenolic compounds in methanol fraction of the extract. Similar results were described by other studies in the literature for other extracts of plants [14]. The *C. opaca* leaves provided us with plentiful of different sorts of polyphenolic compounds as an incredible source of antioxidant, exhibited by the remarkable EC_50_ values in different extracts. The observed differential scavenging activities of the extracts against various systems may be referred to the different mechanisms of the radical antioxidant reactions in the different assays. Hagerman et al. [15] have reported that the high molecular weight phenolics (tannins) have more abilities to quench free radicals (ABTS^·+^) and their effectiveness depends on the molecular weight, the number of aromatic rings and nature of hydroxyl group’s substitution than the specific functional groups. Free radical (ABTS^·+^) scavenging activity of *C. opaca* leaves extracts might be due to the presence of high molecular weight phenolics such as catechin, and rutin derivatives. The *C. opaca* leaves extracts exhibit remarkable H_2_O_2_ and OH· radical scavenging capacity rendering, their utilization in different ailments associated with oxidative stress [16, 17]. Recent investigations have shown that many flavonoids and related polyphenols contribute significantly to the antioxidant activity of medicinal plants. Our results revealed that there is a strong and significant correlation between TPC and DPPH^·^ free radical scavenging activity and H_2_O_2_ scavenging activity for the *C. opaca* leaves extracts, while the other assays have non-significant correlation with the TPC. This could be due to the difference in the stoichiometry of reactions between the antioxidant compounds in the extracts and the various radicals, which may be inferred as a reason for the difference in their scavenging potential. The diversity in radical scavenging shown in these assays may also be due to factors like stereo selectivity of the radicals or the differential solubility that may be justified in case of crude extracts, which contain a variety of antioxidants.

## Materials and methods

### Plant collection and extraction

**Fig. 3:**
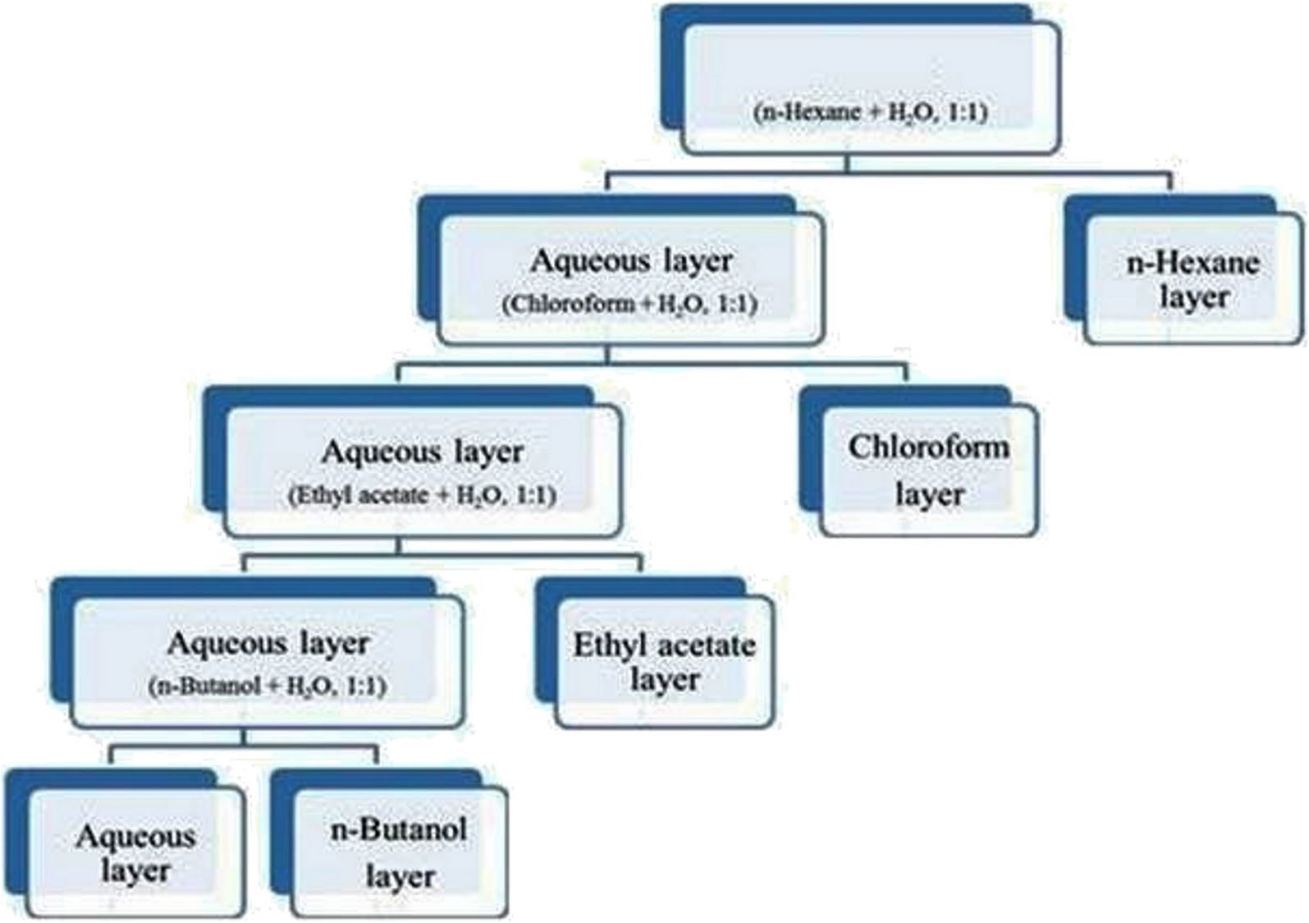
Stepwise extraction procedure

*Carrisa opaca* leaves at maturity were collected from Islamabad, identified and a specimen was submitted at Herbarium of Pakistan, Quaid-I-Azam University Islamabad. Leaves were shades dried at room temperature for two weeks, chopped and ground mechanically. 1.5 kg of dried sample was extracted twice with 5 l of 70% methanol at 25 °C for 48 h. The extracts were filtrated through Whatman No. 1 filter paper and combined followed by concentration using a rotary evaporator (Panchun Scientific Co., Kaohsiung, Taiwan) under reduced pressure at 40 °C. After crude extraction it was further fractionated with solvent extraction to various fractions (Fig. 3). Each of the fractions obtained were dried using a rotary evaporator. The dry extract obtained with each solvent was weighed. The percentage yield was expressed in terms of air dried weight of plant material.

### Total phenolic and flavonoids contents

The total phenolic content was determined using the method [18] with certain modifications. Calibration curve was prepared by mixing methanolic solution of gallic acid (1 ml; 0.025–0.400 mg/ml) with 5 ml Folin–Ciocalteu reagent (diluted tenfold) while total flavonoids content was determined by using a method described [13]. All fractions were run in triplicate.

### In vitro antioxidant activity

The free-radical scavenging activity of the various fractions, gallic acid and ascorbic acid was measured with the stable radical diphenylpicrylhydrazyl (DPPH) in terms of hydrogen-donating or radical-scavenging activity [19] with some modifications. ABTS assay was performed according to the protocol [20] while superoxide scavenging was determined by the nitroblue tetrazolium reduction method [21]. The scavenging capacity for hydrogen peroxide was measured according to the method [22]. The effect of extracts on hydroxyl radicals was assayed by using the deoxyribose method [23]. The extracts were assessed for their ability to compete with ferrozine for iron (II) ions in free solution. The chelating ability of ferrous ions by various fractions was estimated by the method [24].

### Statistical analysis

EC_50_ was carried out using graph prism pad software. Experimental results were further analyzed for Pearson correlation coefficient between TPCs, flavonoids and different antioxidant assays and tested for significance by Student’s *t* test (*P* < 0.05). SPSS ver. 14.0 (Chicago, IL, USA) and Microsoft Excel 2007 (Roselle, IL, USA) were used for the statistical and graphical evaluations.

## Conclusion

This study revealed that the activities are may be due to the presence of bioactive phenolic and flavonoid contents.
